# Autoantibodies against p53, MMP-7, and Hsp70 as Potential Biomarkers for Detection of Nonmelanoma Skin Cancers

**DOI:** 10.1155/2021/5592693

**Published:** 2021-07-10

**Authors:** Shi-Han Yang, Can-Tong Liu, Chao-Qun Hong, Ze-Yuan Huang, Huan-Zhu Wang, Lai-Feng Wei, Yi-Wei Lin, Hai-Peng Guo, Yu-Hui Peng, Yi-Wei Xu

**Affiliations:** ^1^Department of Dermatology and Venereology, Affiliated Shantou Hospital of Sun Yat-sen University, 114 Waima Road, Shantou 515041, China; ^2^Department of Clinical Laboratory Medicine, The Cancer Hospital of Shantou University Medical College, 7 Raoping Road, Shantou 515041, China; ^3^Precision Medicine Research Center, Shantou University Medical College, 22 Xinling Road, Shantou 515041, China; ^4^Department of Oncological Laboratory Research, The Cancer Hospital of Shantou University Medical College, 7 Raoping Road, Shantou 515041, China; ^5^Department of Head and Neck Surgery, The Cancer Hospital of Shantou University Medical College, 7 Raoping Road, Shantou 515041, China; ^6^Guangdong Esophageal Cancer Research Institute, Shantou University Medical College, 22 Xinling Road, Shantou 515041, China

## Abstract

Basal cell carcinoma (BCC) and squamous cell carcinoma (SCC) are two predominant histological types of nonmelanoma skin cancer (NMSC), lacking effective early diagnostic markers. In this study, we assessed the diagnostic value of autoantibodies against p53, MMP-7, and Hsp70 in skin SCC and BCC. ELISA was performed to detect levels of autoantibodies in sera from 101 NMSC patients and 102 normal controls, who were recruited from the Cancer Hospital of Shantou University Medical College. A receiver operator characteristic curve was used to evaluate the diagnostic value. The serum levels of autoantibodies against p53, MMP-7, and Hsp70 were higher in NMSCs than those in the normal controls (all *P* < 0.01). The AUC of the three-autoantibody panel was 0.841 (95% CI: 0.788-0.894) with the sensitivity and specificity of 60.40% and 91.20% when differentiating NMSCs from normal controls. Furthermore, measurement of this panel could differentiate early-stage skin cancer patients from normal controls (AUC: 0.851; 95% CI: 0.793-0.908). Data from Oncomine showed that the level of p53 mRNA was elevated in BCC (*P* < 0.05), and the Hsp70 mRNA was upregulated in SCC (*P* < 0.001). This serum three-autoantibody panel might function in assisting the early diagnosis of NMSC.

## 1. Introduction

Skin cancer is the most common type of carcinoma, categorized as melanoma and nonmelanoma skin cancer (NMSC). Except for the Merkel cell carcinoma, basal cell carcinoma (BCC) and squamous cell carcinoma (SCC) are the other two predominant histological types of NMSC [1]. Skin BCC, originating from cells in the interfollicular epidermis or upper infundibulum, accounted for about 80% of NMSCs [2]. Compared with the general population, patients with a history of skin BCC had a 3-year cumulative risk of 44% and a tenfold increase in recurrence [3, 4]. As the second most common NMSC, patients with early-stage skin SCC have 5-year disease-specific survival of more than 75%. However, for those with lymph node metastasis, the 5-year overall survival is lower than 50%, and it is mostly lower than 10% when distant metastasis happens [5]. The diagnosis of skin cancer is mainly based on pathology; thus, it is necessary to explore some serological indicators to assist the diagnosis.

Tumor-associated antigens (TAAs) can not only be overexpressed in cancer tissues but also be secreted into peripheral blood, which might be the basis of distant metastasis [6]. After recognizing TAAs, the immune system can secrete specific autoantibodies [7]. In the last decade, numerous studies focused on the diagnostic value of serum autoantibodies against TAAs in different cancers, especially in the early detection [8–11]. When it came to skin cancers, most of the related research paid attention to melanoma, but not NMSC [12–14]. Whether serum autoantibodies have a potential diagnostic value for NMSC needs to be addressed.

In this study, we assessed the diagnostic value of an autoantibody panel against three well-recognized TAAs in skin SCC and BCC, including p53, matrix metalloproteinase-7 (MMP-7), and heat shock protein 70 (Hsp7). Encoded from TP53, a tumor-suppresser gene, p53 was described as the first antigen to elicit autoantibodies in cancer [15]. From then on, there was an increase in research about autoantibodies against p53 in cancers, such as lung [16], esophageal [17], and oral cancer [18]. Autoantibodies against matrix metalloproteinase-7 (MMP-7), a member of the matrix metalloproteinase family, were also found in many cancers, including esophageal [17] and oral squamous cell carcinoma [19]. The last antigen, Hsp70, a highly immunogenic antigen, has been shown to induce an autoantibody response in several cancers [20].

## 2. Materials and Methods

### 2.1. Experimental Subjects

Serum samples were collected from participants diagnosed pathologically with skin cancers, who were recruited from the Cancer Hospital of Shantou University Medical College between November 2014 and January 2020. The noneligible skin cancer standards for the study were set as follows: tumors with any other pathological types including melanoma and dermatofibrosarcoma or without concise pathological types and patients who suffered from other malignancies before. Normal controls were comprised of healthy volunteers who had evidence of tumor inexistence. Approval from the Institutional Ethics Committee of the Cancer Hospital of Shantou University Medical College and written consent from each participant was obtained. The study conformed to the provisions of the Declaration of Helsinki.

The clinicopathological data of these participants were also collected for further study, including age, gender, histological type, site of tumor, and TNM stage. TNM stage was evaluated according to the 8th edition published by the Union for International Cancer Control for skin cancer staging [21]. In the present study, tumors with stage I or II were defined as early-stage skin cancers, and tumors with stage III or IV were considered an advanced stage. Peripheral blood samples were collected before anticancer treatments, clotted at room temperature for 30 min, and centrifuged at 1250 g for 5 min. Then, the sera were removed and stored at -80°C in the biobank until the experiment began.

### 2.2. Recombinant Protein Expression

The coding sequence regions for P53 (NM_001276760.1), MMP-7 (NM_002423.3), and HSP70 (NM_005345.5) were subcloned into the pDEST17 expression vector (Invitrogen, Waltham, MA). The expression, purification, and analysis of these recombinant proteins were conducted as described in our previous studies [17].

### 2.3. Enzyme-Linked Immunosorbent Assay (ELISA)

ELISA was performed as previously described by SH Yang and CT Liu, who were blinded to all patient information [17]. Briefly, purified recombinant antigens of p53, MMP-7, and Hsp70 were diluted in 50 mM bicarbonate buffer. Serum samples and quality control samples (a pooled serum sample collected randomly from 50 cancer patients) were diluted at 1 : 110 in blocking buffer and were then incubated at 37°C for one hour, as well as the control rabbit polyclonal antibodies. After washing for three times, horseradish peroxidase-conjugated goat anti-rabbit IgG or anti-human IgG (Santa Cruz Biotechnology, Santa Cruz, CA) was added as the secondary antibody. After one-hour incubation and washing again, 3,3′,5,5′-tetramethylbenzidine (TMB) and hydrogen peroxide were added for color formation. The absorbance of each well was read at 450 nm and referenced to 630 nm by a plate microplate reader (Thermo Fisher Scientific, Boston, USA) in five minutes. All serum sample detections were conducted in duplicate.

### 2.4. Oncomine Database Analysis

We obtained RNA-sequencing data of p53, MMP-7, and Hsp70 gene expression from Oncomine, which were comprised of 15 skin BCC samples, 11 skin SCC samples, and 4 normal samples [22]. The heat map and box plot were conducted to compare their expressions.

### 2.5. Statistical Analysis

All analyses were produced with IBM SPSS Statistics 24 (SPSS Inc., Chicago, IL, USA), GraphPad Prism 8.0 (GraphPad Software, San Diego, CA, USA), and R package 3.6.1. The Mann–Whitney *U* test was used for comparing the level of different markers between the normal group and the skin cancer group and between the SCC group and the BCC group. We applied logistic regression analysis to construct a predicted probability (*P*) of being diagnosed with skin cancer as one marker from the three autoantibodies. Receiver operating characteristic (ROC) analysis was performed to assess the diagnostic boundaries including the area under the ROC curve (AUC) with the 95% confidence interval (CI). The maximum sensitivity was achieved when the specificity was set at more than 90%. For the clinical diagnosis practice of tumor markers, the sensitivities with 95% specificities were also shown. We compared positive rates of the autoantibody panel before and after surgical resection in skin cancer patients with Fisher's exact test. The pheatmap R package was applied to plot the expression heat map of mRNAs in different patients. In this study, *P* < 0.05 in two tails was seen as statistically significant.

## 3. Results

### 3.1. Patient Characteristics

**Figure 1:**
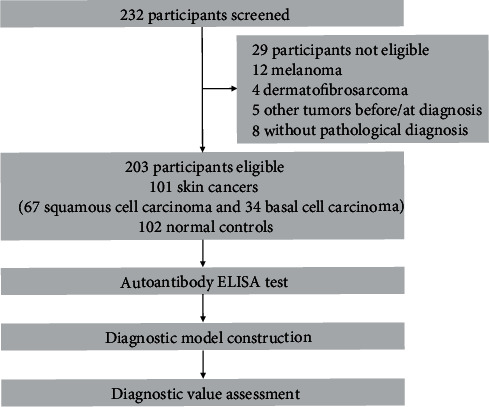
Study profile. ELISA: enzyme-linked immunosorbent assay.

As shown in Figure 1, after eliminating 29 patients including 12 with melanoma, 4 with dermatofibrosarcoma, 5 with other tumors when being diagnosed or before diagnosis, and 8 without pathological diagnosis, 101 NMSCs and 102 normal controls were recruited into our study. There were 67 patients with SCC (66.3%) and 34 patients with BCC (33.7%) in this NMSC group. Patients with stages I and II accounted for 33/101 and 36/101, respectively (Table 1).

### 3.2. Serum Autoantibody Level

**Figure 2:**
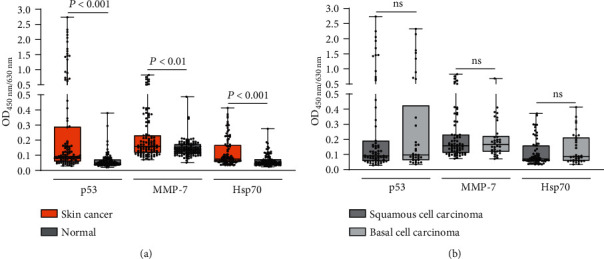
Serum autoantibody measurements in study subjects. (a) The OD values of autoantibodies against p53, MMP-7, and Hsp70 were detected from patients with NMSCs and normal controls. (b) The levels of autoantibodies against p53, MMP-7, and Hsp70 were shown in squamous cell carcinoma and basal cell carcinoma. ns: no statistical significance.

As illustrated in Figure 2(a), the levels of these three autoantibodies were statistically higher in the skin cancer groups than those in the normal groups (p53 and Hsp70: both *P* < 0.001; MMP-7: *P* < 0.01). In order to define the specificity in distinguishing SCC and BCC, we made a comparison of these autoantibody levels between SCC and BCC. However, there was no statistical significance between them (Figure 2(b)).

### 3.3. The Three-Autoantibody Panel for Detection of NMSC

**Figure 3:**
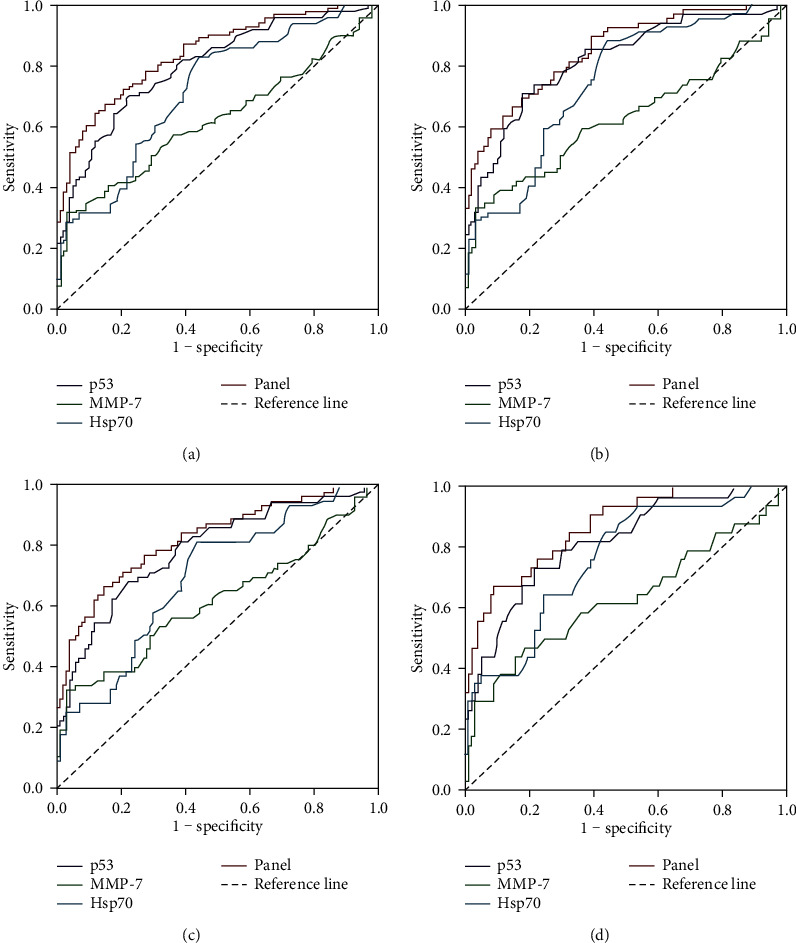
Diagnostic performance of the individual autoantibodies and the autoantibody panel in the detection of skin cancer. (a) Receiver operating characteristic (ROC) curves for the ability to distinguish individuals with skin cancer from normal controls. (b) ROC curves for the ability to distinguish individuals with early-stage skin cancer from normal controls. (c) ROC curves for the ability to distinguish patients with squamous cell carcinoma from normal controls. (d) ROC curves for the ability to distinguish patients with basal cell carcinoma from normal controls.

Autoantibodies against p53, MMP-7, and Hsp70 were all identified to be valid predictors, with the *P* value calculated by ln[*P*/(1 − *P*)] = 10.347 × (p53) + 5.590 × (MMP‐7) + 9.483 × (Hsp70) − 2.828. Then, the *P* value was applied to draw the ROC curve. From ROC analysis (Figure 3), with the specificity of over 90%, autoantibodies against p53 acquired an AUC value of 0.799 (95% CI: 0.739-0.859) in distinguishing NMSCs from normal controls with the sensitivity of 48.5% (Table 2), while the AUC of autoantibodies against MMP-7 and Hsp70 was 0.621 and 0.719, respectively, with sensitivities of 34.7% and 31.7%. When combining these three biomarkers into a panel, we got the AUC value of the panel improving to 0.841 (95% CI: 0.788-0.894) with the sensitivity of 60.4% and the specificity of 91.2% when differentiating the NMSC group from the normal control group. Furthermore, this three-autoantibody panel had a great AUC (0.851, 95% CI: 0.793-0.908) and moderate sensitivity (59.4%) to diagnose early-stage NMSC patients. Great diagnostic values were also observed in different histological types. Meanwhile, we assessed the diagnostic performance of this panel when the specificity was set at 95.0%, and we found that the sensitivities of our panel remained high for the diagnosis of NMSC and early-stage NMSC (Table 2). These results indicated the robustness of this four-autoantibody panel. To conclude, this panel might be a potential biomarker for distinguishing NMSC patients from normal controls.

### 3.4. Level of the Three-Autoantibody Panel after Surgical Treatment

**Figure 4:**
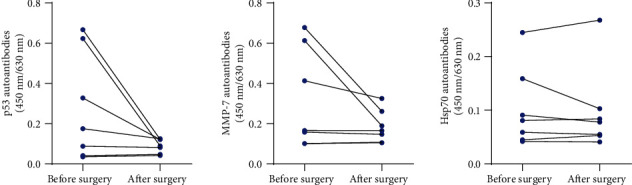
Levels of individual autoantibodies in patients with skin cancer after surgical resection.

We selected 7 NMSC patients with positive results of the three-autoantibody panel before surgery, and we compared the changes in postoperative levels at four weeks. The results showed that the panel became negative at postoperation in 5 cases (5/7, *P* < 0.05). As shown in Figure 4, the levels of autoantibodies against p53 and MMP-7 showed a descending trend after surgical resection of primary tumors, compared with the corresponding preoperative samples. However, for the Hsp70 autoantibody, it seems that the levels did not change after surgery (Figure 4). There is a great need to further explore the monitoring treatment effect of the single autoantibody or the panel for NMSC in a large sample set.

### 3.5. Correlation between Autoantibodies and Clinicopathological Variables

**Figure 5:**
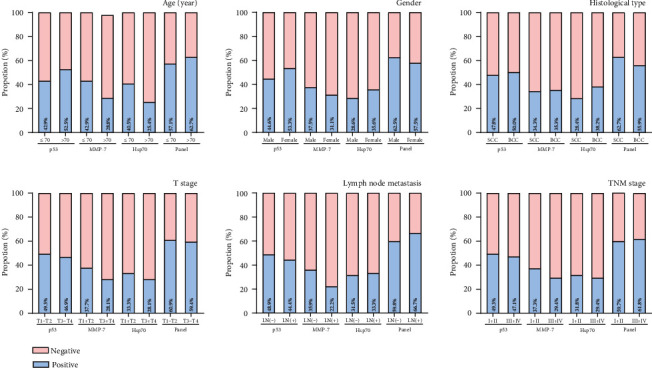
Rate of positive results for p53, MMP-7, Hsp70, and the panel in patients with NMSC according to the clinicopathological variables of tumors.

We next evaluated the relationship of the serum levels of autoantibodies against p53, MMP-7, and Hsp70 with clinicopathological features in NMSCs (Figure 5). However, there were no significant associations between the serum levels of autoantibodies with clinicopathological variables examined, including age, gender, histological types, invasion depth, lymph node status, and early- and advanced-stage groups (all *P* > 0.05).

### 3.6. mRNA Levels of p53, MMP-7, and Hsp70 from Oncomine

**Figure 6:**
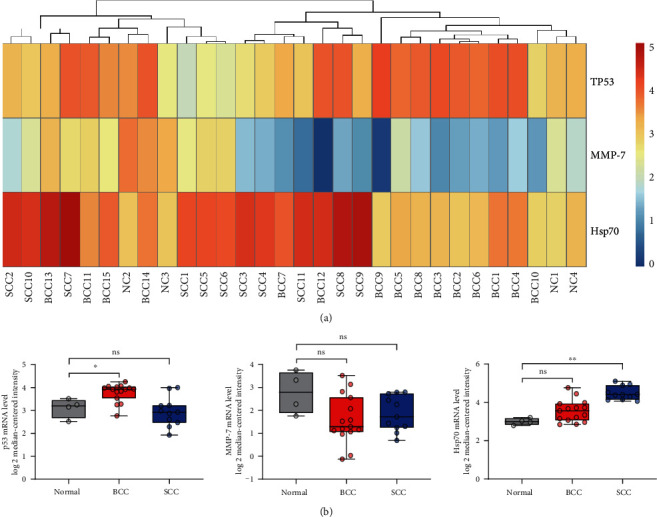
RNA-sequencing data of gene expression of p53, MMP-7, and Hsp70 from Oncomine in SCC, BCC, and corresponding normal tissues. (a) Hierarchical clustering of mRNA expression level of p53, MMP-7, and Hsp70 between patients with skin cancer and controls. (b) The combination of scatter plots and box plots to exhibit the mRNA expression level of p53, MMP-7, and Hsp70 between patients with skin cancer and controls. *P* values were analyzed by Students' *t*-test. ^∗^*P* < 0.05; ^∗∗^*P* < 0.001. ns: no statistical significance; NC: normal control; BCC: basal cell carcinoma; SCC: squamous cell carcinoma.

In order to further evaluate the levels of mRNA of p53, MMP-7, and Hsp70, we explored the datasets of skin SCC and BCC in Oncomine, and the levels of mRNA in tissues were visually shown in the heat map (Figure 6(a)). As exhibited in Figure 6(b), the p53 mRNA level was significantly higher in BCC than in normal skin (*P* < 0.05), while the Hsp70 mRNA level was higher in SCC (*P* < 0.001). However, there was no significant difference of the MMP-7 mRNA level between BCC or SCC and normal skin.

## 4. Discussion

In this study, we conducted a three-autoantibody panel to evaluate its diagnostic value in NMSC, including skin SCC and BCC. As the results showed, the serum levels of autoantibodies against p53, MMP-7, and Hsp70 were all higher in NMSC patients than in normal controls. ROC curve illustrated that the AUC of the three-autoantibody panel was larger than that of a single autoantibody, and the meaningful diagnostic efficacy of the autoantibody panel in early-stage tumors could also be found. Through exploring the RNA-sequence data in Oncomine, we found that p53 and Hsp70 mRNAs were upregulated in BCC and SCC, respectively.

Autoantibodies against p53 have been detected as over expression in several tumors [16–18]. In our study, the level of autoantibodies against p53 was higher in NMSC when compared with that of the normal controls. However, Moch et al. found the discrepant result that there was no difference of the level of p53 autoantibodies between skin cancers and normal controls [23]. The AUC of the p53 autoantibody in our study was 0.799 (95% CI: 0.739-0.859) in NMSCs and 0.819 (95% CI: 0.754-0.885) in early-stage NMSCs with a slightly improved sensitivity of 49.3% compared with the former (48.50%), indicating the potential value of anti-p53 in distinguishing NMSCs from normal controls. Accumulating evidence highlighted serum p53 autoantibodies as a broad-spectrum biomarker in early detection of cancer, with the AUCs ranging from 0.627 to 0.781 [8, 24–28]. A recent study including 182 stage 0/I breast cancer patients showed a positive rate of 10% for the p53 autoantibody [29]. Another study with the use of a new electrochemiluminescence immunoassay indicated that p53 autoantibodies were helpful in detecting esophageal and colorectal cancers with high specificity [30]. As we know, there were some researches about the diagnostic value of autoantibodies against MMP-7 and Hsp70 in other cancers but not in skin cancers. Jiang et al. found that the sensitivity of MMP-7 autoantibodies for diagnosing early-stage oral cancers was 48.6% and the AUC was 0.761 [19]. In the upper gastrointestinal neoplasms, the AUC of MMP-7 autoantibodies was varied from 0.597 to 0.870 [23, 31]. Zhong et al. found an improved level of serum Hsp70 autoantibodies in non-small-cell lung cancer patients with a diagnostic AUC of 0.731, a sensitivity of 0.74, and a specificity of 0.73 [32]. It was reported that the AUCs of autoantibodies against Hsp70 were 0.652 in esophagogastric junction cancer [24] and 0.765 in nasopharyngeal carcinoma [27]. These similar diagnostic values were found in our study. Thus, although our study showed the diagnostic value of any single autoantibody, these autoantibodies were not limited to NMSCs.

A single autoantibody might lack plenty of sensitivity and specificity for diagnosis, while a panel of autoantibodies might overwhelm this problem [33, 34]. Identification of new serum biomarkers for cancer diagnosis is an important means to improve clinical efficacy, especially for the early-stage malignancies [35]. Previous reports of other tumors have highlighted the potential value of autoantibody panels [17, 24]. For example, in our previous research, the AUC of autoantibodies against a panel of six TAAs (p53, NYESO-1, PRDX6, MMP-7, Hsp70, and BMI-1) might improve to 0.786 with a sensitivity of 50.0% and a specificity of 90.5% in the diagnosis of early-stage esophagogastric junction adenocarcinoma when compared with the individuals [24]. There were less studies published about the early diagnostic value of an autoantibody panel in NMSCs. Herein, our study might be the first one to explore the diagnostic value of a panel of autoantibodies against p53, MMP-7, and Hsp70 in the skin SCC and BCC. In early-stage SCC and BCC of this study, the AUC and sensitivity of the panel showed great discrimination from normal controls (AUC: 0.851 (95% CI: 0.793-0.908); sensitivity: 59.40%), which indicated that this panel might be the potential early detection marker of NMSCs in future clinical practices.

Autoantibodies are secreted by the immune system after they have recognized antigens, which might be produced by abnormal transcription or modification of a gene, like histone acetylation [36]. The synchronous increases of autoantibodies and mRNAs were observed in several cancers, for instance, BMI-1 in cervical carcinoma [37] and FKBP52 in breast cancer [38]. In skin cancers, some related researches were also conducted. Moch et al. found the presence of a mutation of the p53 gene in skin BCC and SCC, but the low levels of serum p53 autoantibodies were detected [23]. Unlike these publications, in our study, we found that the levels of p53 autoantibodies were high both in skin BCC and SCC (*P* < 0.001), and data from Oncomine showed that the p53 mRNA level was significantly higher in BCC than in normal skin (*P* < 0.05). It could be suspected that the inconsistency might be due to the different races, and the production of p53 autoantibodies might not be associated with gene mutation in skin BCC. As for Hsp70, data from Oncomine showed the upregulated Hsp70 mRNA level in skin SCC (*P* < 0.001). Wood et al. observed the overexpression of Hsp70 autoantigens in skin SCC [39], and our study also found a higher level of Hsp70 autoantibodies of skin SCC when compared with that of normal participants (*P* < 0.001), which might indicate the vital role of Hsp70 in the tumorigenesis of skin SCC. Recently, two studies showed that Hsp70 and Hsp90 are beneficial to the reverse regulation of p53 conformation and activity in the cell environment. Through the cycle of the molecular chaperone mechanism, p53 is constantly reconstituted, and its active state is related to the stress state of cells, so that p53 can make a rapid response to the changes of the cell environment to prevent irreversible degradation in an extreme environment [40, 41]. Based on the upregulation of p53 mRNA in BCC and Hsp70 mRNA in SCC compared with normal skin, we speculated that the different status of p53 driven by Hsp70 and Hsp90 might hint of the different histological types of NMSC. Thus, the association between p53 and Hsp70 expression levels and the induction of their autoantibodies in NMSC, especially skin SCC and BCC, needs to be further validated in a future study.

Mitsui et al. had observed that the upregulation of MMP-7 mRNA in skin invasive SCC driven by IL-24 might enhance the proliferation, migration, and invasion of skin SCC cells [42]. However, in this study, the public data from Oncomine showed that there was no significant difference in the expression of MMP-7 mRNA between skin cancers and normal skins. This inconsistency might result from the small sample size of the obtained data. Thus, whether the mechanism of the production of the MMP-7 autoantibody is correlated to its high expression of gene needs to be further explored.

There were several important shortcomings that we were unable to control. First, as the sample size was small from Oncomine, it would be better to collect the related skin cancer tissues and normal tissues of our participants to further evaluate p53, MMP-7, and Hsp70 mRNA expression. However, we failed to finish this for the reason that some of the enrolled patients rejected surgery when making treatment decisions. Second, our research was a single-center and small-sample one which needs to be further validated in a future study. Third, as exposure to ultraviolet B irradiation is widely seen as a risk factor of skin cancers [43], it would be better to compare the relationship of excessive exposure to sunshine and our autoantibody panel. If this work was completed in the future, it would further supplement the mechanism of skin tumorigenesis and ultraviolet B irradiation. Finally, due to the lack of follow-up data, we were unable to assess the prediction value of these autoantibodies in the prognosis of skin SCC and BCC. Actually, whether tumor-associated autoantibodies could be used as prognostic markers is still controversial and needs further clarification. Autoantibody against p53 has most extensively been studied with regard to its prognostic value. Most studies suggested that autoantibodies against p53 might be associated with tumor prognosis, like ovarian and esophageal cancers [44, 45]. On the contrary, several studies showed that the prognostic value of the p53 autoantibody for the overall survival prediction is limited [46]. For autoantibodies against MMP-7 and Hsp70, there are relatively few studies on tumor prognosis. Only one study indicated that the MMP-7 autoantibody might be a novel prognostic biomarker for oral squamous cell carcinoma [19]. A few studies found that positivity for the Hsp70 autoantibody tended to have a worse prognosis in cancer patients [47]. Based on the abovementioned evidences, we suppose that such autoantibodies might be associated with the prognosis with NMSC, which needs to be verified in future work.

## 5. Conclusions

In summary, we utilized ELISA to detect the serum levels of three autoantibodies in patients with skin SCC and BCC and compared it with normal controls. The high levels were observed, as well as the high diagnostic value, especially in early-stage skin cancers. The RNA-seq public data from Oncomine revealed significant upregulation of p53 mRNA in BCC and Hsp70 mRNA in SCC. Taken together, our current findings could benefit future studies in targeting this serum panel for the screening of NMSC.

## Figures and Tables

**Table 1:** Details of the NMSC patients and normal controls.

Test parameter	Category	NMSC (*n* = 101)		Normal (*n* = 102)	
		Cases	%	Cases	%
Gender	Male	56	55.4%	50	49.0%
	Female	45	44.6%	52	51.0%

Age at enrollment (years)	≤60	16	15.8%	—	—
	61-70	26	25.7%	—	—
	71-80	37	36.6%	—	—
	81-100	22	21.8%	—	—

Histological type	Squamous cell carcinoma	67	66.3%	—	—
	Basal cell carcinoma	34	33.7%	—	—

Site of tumor	Face/scalp/forehead	50	49.5%	—	—
	Nose/ear/lip/eyelid	31	30.7%	—	—
	Leg/arm/hand	10	9.9%	—	—
	Others	10	9.9%	—	—

T category	T1	32	31.7%	—	—
	T2	37	36.6%	—	—
	T3	21	20.8%	—	—
	T4	11	10.9%	—	—

Lymph node metastasis	Positive	9	8.9%	—	—
	Negative	92	91.1%	—	—

TNM stage	I	33	32.7%	—	—
	II	36	35.6%	—	—
	III	17	16.8%	—	—
	IV	15	14.9%	—	—

Note: NMSC: nonmelanoma skin cancer.

**Table 2:** Diagnostic values for the individual autoantibodies and the autoantibody panel in NMSC.

	AUC	SEN	SPE	PPV	NPV	PLR	NLR
*All stages of NMSC*							
p53 autoantibody	0.799 (0.739-0.859)	48.5%/40.6%	90.2%/95.0%	83.1%/89.2%	63.8%/61.7%	4.95/8.29	0.57/0.63
MMP-7 autoantibody	0.621 (0.543-0.699)	34.7%/31.7%	91.2%/95.0%	79.6%/86.5%	58.5%/58.4%	3.94/6.47	0.72/0.72
Hsp70 autoantibody	0.719 (0.649-0.789)	31.7%/29.7%	93.1%/95.0%	82.0%/85.7%	57.9%/57.7%	4.59/6.06	0.73/0.74
The autoantibody panel	0.841 (0.788-0.894)	60.4%/51.5%	91.2%/95.0%	87.2%/91.2%	69.9%/66.4%	6.86/10.51	0.43/0.51
*Early-stage of NMSC*							
p53 autoantibody	0.819 (0.754-0.885)	49.3%/43.5%	90.2%/95.0%	77.3%/85.8%	72.4%/70.3%	5.03/8.88	0.56/0.59
MMP-7 autoantibody	0.627 (0.536-0.718)	37.7%/33.3%	91.2%/95.0%	74.4%/82.2%	68.4%/67.8%	4.28/6.80	0.68/0.70
Hsp70 autoantibody	0.746 (0.673-0.820)	31.9%/30.4%	93.1%/95.0%	75.8%/80.8%	66.9%/66.8%	4.62/6.20	0.73/0.73
The autoantibody panel	0.851 (0.793-0.908)	59.4%/52.2%	91.2%/95.0%	82.1%/87.8%	76.8%/74.6%	6.75/10.65	0.45/0.50
*Skin squamous cell carcinoma*							
p53 autoantibody	0.790 (0.719-0.860)	47.8%/38.8%	90.2%/95.0%	76.2%/83.8%	72.5%/70.3%	4.88/7.92	0.58/0.64
MMP-7 autoantibody	0.616 (0.524-0.708)	34.3%/32.8%	91.2%/95.0%	71.8%/81.4%	67.9%/68.3%	3.90/6.69	0.72/0.71
Hsp70 autoantibody	0.697 (0.616-0.777)	28.4%/25.4%	93.1%/95.0%	73.0%/77.3%	66.5%/66.0%	4.12/5.18	0.77/0.78
The autoantibody panel	0.826 (0.761-0.891)	56.7%/49.3%	91.2%/95.0%	80.9%/86.8%	76.3%/74.1%	6.44/10.06	0.48/0.53
*Skin basal cell carcinoma*							
p53 autoantibody	0.818 (0.736-0.900)	50.0%/44.1%	90.2%/95.0%	63.0%/85.5%	84.4%/72.2%	5.10/9.00	0.55/0.59
MMP-7 autoantibody	0.631 (0.509-0.753)	35.3%/29.4%	91.2%/95.0%	57.2%/79.7%	80.9%/67.3%	4.01/6.00	0.71/0.74
Hsp70 autoantibody	0.763 (0.672-0.854)	38.2%/38.2%	93.1%/95.0%	64.9%/83.6%	81.9%/70.1%	5.54/7.80	0.66/0.65
The autoantibody panel	0.870 (0.803-0.937)	67.6%/55.9%	91.2%/95.0%	71.9%/88.2%	89.4%/76.7%	7.68/11.41	0.35/0.46

Note: data after “/” were obtained at a specificity of 95.0%. NMSC: nonmelanoma skin cancer; AUC: the area under the receiver operating characteristic curve; SEN: sensitivity; SPE: specificity; PPV: positive predictive value; NPV: negative predictive value; PLR: positive likelihood ratio; NLR: negative likelihood ratio.

## Data Availability

The data used to support the findings of this study are available from the corresponding authors upon request.
